# Chronic Inflammation’s Transformation to Cancer: A Nanotherapeutic Paradigm

**DOI:** 10.3390/molecules28114413

**Published:** 2023-05-29

**Authors:** Sayed Sartaj Sohrab, Riya Raj, Amka Nagar, Susan Hawthorne, Ana Cláudia Paiva-Santos, Mohammad Amjad Kamal, Mai M. El-Daly, Esam I. Azhar, Ankur Sharma

**Affiliations:** 1 Special Infectious Agents Unit, King Fahd Medical Research Center, King Abdulaziz University, Jeddah 21589, Saudi Arabia; ssohrab@kau.edu.sa (S.S.S.);; 2 Department of Medical Laboratory Sciences, Faculty of Applied Medical Sciences, King Abdulaziz University, Jeddah 21589, Saudi Arabia; 3 Department of Biochemistry, Bangalore University, Banglore 560056, India; 4 Department of Life Science, School of Basic Science and Research, Sharda University, Greater Noida 201310, India; 5 School of Pharmacy and Pharmaceutical Sciences, Ulster University, Coleraine BT52 1SA, UK; 6 Department of Pharmaceutical Technology, Faculty of Pharmacy of University of Coimbra, University of Coimbra, 3000-548 Coimbra, Portugal; 7 LAQV, REQUIMTE, Department of Pharmaceutical Technology, Faculty of Pharmacy of University of Coimbra, University of Coimbra, 3000-548 Coimbra, Portugal; 8 Enzymoics Inc., Hebersham, NSW 2770, Australia; 9 Novel Global Community Educational Foundation, Hebersham, NSW 2770, Australia; 10 Strathclyde Institute of Pharmaceutical and Biomedical Sciences, University of Strathclyde, Glasgow G1 0RE, UK

**Keywords:** cancer, inflammation, nanoparticles, drug delivery, inflammatory pathways

## Abstract

The body’s normal immune response against any invading pathogen that causes infection in the body results in inflammation. The sudden transformation in inflammation leads to the rise of inflammatory diseases such as chronic inflammatory bowel disease, autoimmune disorders, and colorectal cancer (different types of cancer develop at the site of chronic infection and inflammation). Inflammation results in two ways: short-term inflammation i.e., non-specific, involves the action of various immune cells; the other results in long-term reactions lasting for months or years. It is specific and causes angiogenesis, fibrosis, tissue destruction, and cancer progression at the site of inflammation. Cancer progression relies on the interaction between the host microenvironment and tumor cells along with the inflammatory responses, fibroblast, and vascular cells. The two pathways that have been identified connecting inflammation and cancer are the extrinsic and intrinsic pathways. Both have their own specific role in linking inflammation to cancer, involving various transcription factors such as Nuclear factor kappa B, Activator of transcription, Single transducer, and Hypoxia-inducible factor, which in turn regulates the inflammatory responses via Soluble mediators cytokines (such as Interleukin-6, Hematopoietin-1/Erythropoietin, and tumor necrosis factor), chemokines (such as Cyclooxygenase-2, C-X-C Motif chemokines ligand-8, and IL-8), inflammatory cells, cellular components (such as suppressor cells derived from myeloid, tumor-associated macrophage, and acidophils), and promotes tumorigenesis. The treatment of these chronic inflammatory diseases is challenging and needs early detection and diagnosis. Nanotechnology is a booming field nowadays for its rapid action and easy penetration inside the infected destined cells. Nanoparticles are widely classified into different categories based on their different factors and properties such as size, shape, cytotoxicity, and others. Nanoparticles emerged as excellent with highly progressive medical inventions to cure diseases such as cancer, inflammatory diseases, and others. Nanoparticles have shown higher binding capacity with the biomolecules in inflammation reduction and lowers the oxidative stress inside tissue/cells. In this review, we have overall discussed inflammatory pathways that link inflammation to cancer, major inflammatory diseases, and the potent action of nanoparticles in chronic inflammation-related diseases.

## 1. Introduction

**Figure 1:**
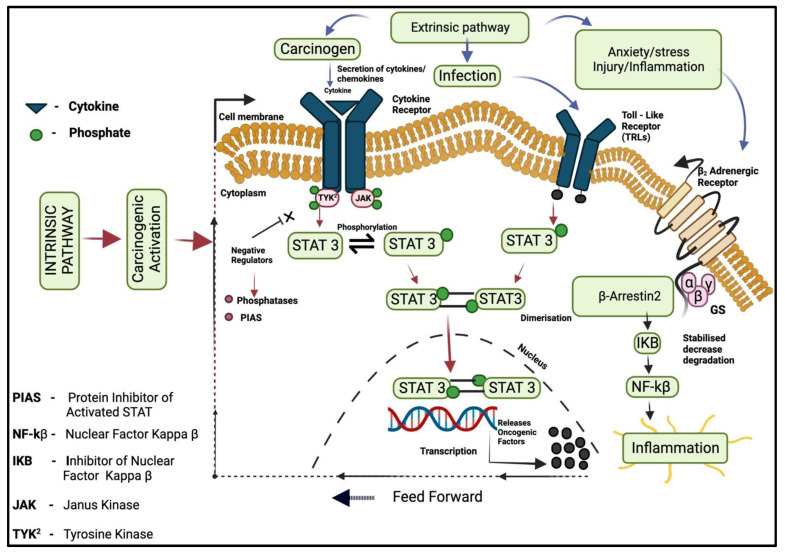
The cascades of inflammatory pathways leading to the onset of cancer, Protein inhibitor of activated STAT (PIAS), NF-kβ (Nuclear Factor Kappa β), IKB (Inhibitor of Nuclear Factor Kappa β), JAK (Janus Kinase), and TYK^2^ (Tyrosine Kinase).

Cancer is the main leading cause of most deaths worldwide nowadays [1]. The connecting relation between cancer and inflammation was observed for the first time in the 19th century by Rudolf Virchow (a German scientist from Wurzburg), who founded the possible indication that inflammation may play a vital part in tumor progression and development. It was mentioned that “lymphoreticular infiltrate” showed the development of tumor cell progression at the site of chronic inflammation [2]. The body’s defense mechanism starts working against a particular internal cell or tissue damage that is caused by a foreign body (such as an irritant, pathogens, or any injury). The body’s WBCs launch a biological response to invade that pathogen as a process of healing [3]. This involves the release of chemicals that trigger the immune system, releases antibodies and proteins, and increases the supply of blood flow to damaged and injured areas. It usually lasts for hours or days in case of certain inflammation (acute). Over a prolonged period, chronic inflammation results in DNA damage and may give a home to cancer development such as Crohn’s disease and Ulcerative colitis. The longer the inflammation lasts, the higher the risk of carcinogenesis. Chronic inflammation is long-term and lasts for years, and it may lead to DNA damage and progress to cancer [4]. Approximately 20% of cancers are linked to infections (hepatitis B and C virus is one of them) [5]. Chronic inflammation is characterized by prolonged cell/tissue injuries, invasion, damage-induced cellular proliferation, tumorigenesis, and metastasis [6]. Chronic inflammation is associated with neoplasia [4]. Inflammation associated with cancer is imposed to fast emergence to resist drugs, showing an attractive targeting strategy to achieve prevention, fast recovery, treatment, and therapy of cancer [7]. The infection that triggers the chronic inflammation increases the chances of cancer risk during injury and progression of infection (Figure 1) (such as helicobacter pylori for hepatocellular carcinoma, mucosal lymphoma and gastric carcinoma, hepatitis virus for liver and cervical cancer, and other inflammatory bowel diseases aligned with colorectal cancer). Chronic inflammation involves various steps in cancer progression such as tumorigenesis, cellular transformation, invasion, angiogenesis, proliferation, and metastasis [8]. The gastrointestinal system is a notable site for a proportion of these linked tumors from the pharynx as many distal cancers emanate chronically in inflamed gastrointestinal tissue [9]. Hepatitis B and C viruses are responsible for approximately 80% of hepatocellular carcinoma cases and human papilloma, the leading cause of anogenital cancer [10]. Macrophage expresses about 10–20% of mononuclear cells found in the lamina of the intestine and plays a vital part in chronic inflammatory responses. Neoplastic cells are capable to allure different types of cells through the secretion of extracellular protease, and cytokinesis by various cells in the tumor microenvironment (TEM) [11]. Interleukin-10 is secreted by macrophages and tumor cells, which inhibits cytotoxic T-cells and suppresses the immune response contrary to the tumor [12]. STAT3 plays a vital role in cell proliferation, signaling, invasion, and angiogenesis, its activation is induced by phosphorylation on Tyr 705 residue. NF-kB regulates differentiation and activation of inflammatory T-cells and mediates the proliferation of cancerous cells, metastasis, angiogenesis, and survival by regulating the expression of the target genes: Bcl2, TNF-α, B-cell lymphoma, XS, XL, XIAP (X-linked, an inhibitor of apoptosis protein 3), and Interleukin 6, IKK (IkB kinase) complex. Catalysis is usually carried out by heterodimeric kinase that consists of two subunits, IKKα and IKKβ, which help in the regulation of NF-kB signaling pathways. IL-1 and TNF-α work to activate NF-kB, creating a feedback loop. Chemokines have the ability to activate leukocytes precisely at a specific site and they may stimulate cells to secret proteolytic enzymes that aid the digestion of extracellular matrix and provides the desired path to inflammatory cells for migration and metastasis [13]. Its persuaded migration includes tumor stroma and Macrophage chemotactic protein (MCP-1). Chronic inflammation is described by infiltration, sustained tissue damage, and dysplasia [6]. Chronic intestinal inflammation causes a mutation in TP53 and other cancer cells inside epithelial cells. Macrophages secrets a high number of bioactive products; these macrophages accompany other leukocytes and give rise to excessive amounts of Reactive oxygen species to fight against infection, continuous damage to tissue, and deleterious cell agents. They may give rise to mutagenic factors such as peroxynitrite (reactive, oxidant), which leads to a mutation in the epithelial and stroma cells when reacting with DNA. TNF-alpha may be released by T-lymphocyte [14].

## 2. Inflammatory Factors Involved in Cancer Transformation

Inflammation, when it became chronic, settles down the body’s normal inflammatory process, creating a favorable environment for the development of cancerous cells. There are numerous signaling pathways that are key contributors to generating epigenetic changes outside and inside the cell. The transformation of normal cells to malignant cancerous cells or neoplasms is a multistep process [15]. Many cancers microenvironments can become chronic inflammatory conditions resulting in chronic infections, consumption of tobacco, alcohol, smoking, obesity, and environmental pollutants that can impact the steps of tumor development [16]. Inflammatory angiogenesis is involved in the genesis of new blood vessels from existing vasculature and contributes to tumor development [17]. NF-kB and STAT3 are regulated by the majority of angiogenic genes such as IL-8 and hypoxia-inducible factor 1 alpha. An activator in these signals, along with depletion in TAM, plays a vital role in the reduction of the angiogenic tumor environment [18].

### 2.1. Macrophages and Denticles Cells

Macrophages consist of closely linked bone marrow cells, blood monocytes, macrophages of tissues, and a constituent part of the mononuclear phagocyte system. Macrophages primarily have three major vital roles, phagocytosis, presentation of antigens, and in immunomodulation by producing different cytokinesis and growth factors [19]. Macrophages help in maintaining and initiating inflammation [20]. The secreted pro-inflammatory mediators have a necessary part in the growth of fibroblasts and blood vessels, and in tissue remodeling and regeneration [21]. The process is activated and deactivated by inflammatory processes such as activating signals i.e., cytokinesis and TNF-α are deactivated by removing mediators and inflammatory effector cells. Interleukin 10 helps in the deactivation of activated macrophages [19]. Macrophages are components of innate immunity derived from the myeloid progenitor cell namely known as the granulocyte-macrophage colony forming unit (GM-CFU) inside bone marrow. Tumor-associated macrophages (TAM) are created by cancerous and stroma cells in the tumor and are enlisted by tumor growth factors and chemokines [22].

TAM promotes cancer development mainly by three mechanisms:

1. By enhancing the angiogenic tumor potential factors such as IL-8, VEGF, and MIF, and by promoting lymph-angiogenesis
2. Progression in the growth of tumor
3. Tumor cell invasion, migration, and intravasation at primary sites JAM, and they act on endothelial cells, further promoting the tumor’s neovascularization [23].

Macrophages contain antigen-presenting cells, immunomodulators, and phagocytosis that play a vital role in the initiation and maintenance of inflammatory functions [24]. Macrophages that infiltrate the tumor parenchyma have an M1 phenotype and M2 phenotype present in the tumor microenvironment [22]. M2 macrophages are characterized by their anti-inflammatory and wound-healing endotype, and they are further divided into different subtypes: the M2a macrophages subtype, which responds to Interleukin-4 and Interleukin-13 during fungal and helminth infections [25]. They express a high level of mannose receptor (CD206) and are the largest secretor of pro-fibrotic factors, such as TGF-β and GF (growth factors), etc. Further, they are involved in the repair of tissues/cells and the healing of wounds at the time of inflammation [26]. M2b is by bacterial LPS and immune complexes; they secrete IL1β, TNF-α, and IL-6 that consists of anti-inflammatory effects. M2c is stimulated by Interleukin-10, glucocorticoids, and TGF-β. M2d macrophages are activated in response to co-stimulation with adenosine ligands and TLR ligands; they express CD206 at a low rate, however, they highly express vascular endothelial growth factor (VEGF) and Interleukin-10 [27]. Dendritic cells have an important part in antigen-specific immunity activation and tolerance maintenance. They develop a connecting link between innate and adaptive immunity [28]. T-cells are stimulated by tumor-associated dendritic cells (TADCs) and these associated cells are different from TAM (tumor-associated macrophages). TADCs are considered poor inducers for effective tumor responses [6]. Dendritic cells are excellent antigen-presenting cells (APC) as they engulf pathogens and present antigens on major histocompatibility complexes.

### 2.2. Proinflammatory Cytokinesis

Inflammatory chemokines and the cytokines that are produced by tumor-associated leukocytes and platelets, participate directly in the progression of malignant cancer. The most basic physiological difference between normal and tumor tissue is that many chemokines and cytokines are induced by hypoxia such as TNF-α, InterLeukin 6, and InterLeukin-1 α and β. They are signaled through type 1 cytokines receptors (CCR1) and are the immune-regulatory cytokinesis that favors inflammation. Inflammatory responses are resolute by the net balance between the anti-inflammatory cytokines and proinflammatory cytokines [29]. Anti-inflammatory cytokines control proinflammatory responses. At the time of chronic inflammation, cytokines can lead to cell malignancy and cell transformation, depending on the tumor microenvironment. Studies found evidence that some cytokines play a key role in promoting and inhibiting cancer [30]. Tumor-promoting cytokines are IL-6, transforming growth factor (TGF-β), TNF-α, and IL-23, they activate STAT proteins, suppress apoptosis, and contribute to EMT [8]. Whereas tumor-inhibiting cytokines including IFN-α, IL-2, and IL-12, inhibit tumor growth and angiogenesis, and expand the functional T-cells and Natural killer cells. Cytokines activate STAT proteins that may increase or decrease inflammation in the case of any viral infection; IFNs trigger and activate STAT 1 and STAT 2. HIF-1 induces inflammation and transcription in genes that process angiogenesis and erythropoiesis; it is a pro-tumorigenic factor that promotes the proliferation of cells and the survival of cancerous cells decreases consumption in VHC-lacking renal carcinoma cells by inhibiting C-Myc.

### 2.3. Tumor Necrosis Factor α

TNF-α is an inflammatory cytokine that participates in regulating various signaling processes such as Nuclear factor kB and C-Jun-N-terminal kinase activation contributes to cell death. JNK and NF-kB helps to describe the cellular outcomes. TNF α signals to TNF receptor 1 and 2, TNF receptor 1 activate pro-inflammatory pathways, and TNF receptor 2 binding to membrane-bound TNF that commence tissue regeneration and immune modulation [31]. As a multifunctional cytokine, it plays diverse cellular events such as differentiation, proliferation, and cell death. An inflammatory cell secretes Tumor Necrosis Factor that may have a role in the process, i.e., associated with inflammation-related carcinogenesis. On the other side, TNF could act as a cancer inhibitor/killer [4]. They are also involved in endogenous tumor promotion as they stimulate the metastasis of cancerous cells. In human cancer, Tumor Necrosis Factor can be found in stroma and malignant cells, lungs, breast, prostate, bladder, and colorectal cancer [32]. In a review, it was found that by up-regulating the levels of prion protein (PrP), TNF-α can contribute to malignant cancer [33]. TNF α promotes the formation of inflammatory cytokines and stimulates the permeability of endothelial cells.

### 2.4. Interleukin

IL-6 plays a vital part in inflammatory responses as it is a pleiotropic cytokine. They are mainly secreted by monocytes. The secretion of InterLeukin 6 by immune cells is an indication of severe infection or major cell/tissue injuries [34]. Their pro-oncogenic effect has been demonstrated in different types of carcinomas including colorectal, breast, and lung. IL-6, along with the proteins of the STAT family, helps to regulate carcinogenic processes, the inhibition of apoptotic processes, and the release of ROS and RNS. IL-2 and 12 poses an anti-cancer effect, and the clinically effective efficacy of gene-edited lymphocyte transfer has been identified in those patients suffering from lung cancer [35]. IL-12 is capable of activating cytotoxic immune cells [36]. They induce immune responses against different cancers. Thymus and bone marrow cells, mast cells, granulocytes, macrophages, and almost all cells of the immune system secret/produce IL-10, and are considered to have a potent anti-inflammatory cytokine [37]. IL-10 is secreted by tumor cells as a tumor infiltratory macrophage. IL-10 consists of both pro and anti-tumoral effects [38]. It inhibits NF-kB signaling pathways when IL 10 binds to the receptors Jak-1 and TyK-2 tyrosine kinase phosphorylate, which allows it to interact with STAT-1, 3, and 5, preferring STAT translocation in the nucleus, inducing targeted gene expression over there [39]. IL-1 receptor antagonist (IL-1Ra), when used to treat metastasis in mouse models, has shown a sudden decrease in tumor development, because of the inhibition of IL-1 action, while those mice that have low IL-1 were resistant to the development of metastasis experimentally [40].

## 3. Inflammatory Signaling Pathways

The development of cancer and its responses are mainly affected by inflammation, by either promoting or suppressing tumor development. Multiple signaling pathways are involved such as JAK2-STAT3 (Janus kinase, signal and activator of transcription), nuclear factor kappa-B, toll-like receptor pathway, Inflammatory factors, cGAS/STING (Protein cyclic GMP-AMP Synthase-Stimulator of Interferon Genes), MAPAK (mitogen-activated protein kinase), PI3K-AKT (phosphatidylinositol-3-kinase), and others.

Two pathways can be described, the intrinsic pathway and the extrinsic pathway.

They both include transcription factors such as Nuclear factor kappa B, STAT3, TNF, CCL2, and CXCL8/IL-8 (chemokines) [41].

### 3.1. Intrinsic Pathway

An intrinsic pathway connects inflammation and cancer genes, genetic changes, or an event that causes neoplasia. They can be accountable for the development of an environment that favors inflammation. In intrinsic pathways, oncogenes, genetic events along with neoplasia transformation trigger the inflammatory cascades [6]. Extrinsic pathways in which chronic inflammation caused by injury, infection, and irritants extensively increase the chance of cancer development. These two pathways converge to activate transcription factors, which results to bring the formation of inflammatory mediators and activate different leukocytes, which gives rise to cancer-related inflammatory microenvironments [42]. The RET/human papillary thyroid carcinoma (PTC) activated transcriptome includes Interleukin-1 beta and COX-2. COX-2 is highly expressed during breast cancer, and they are involved in prostaglandin synthesis. Colony-stimulating factors promote the selection of leukocytes and their survival [43]. In research, it was found that the epidermal growth factor receptor activation in the glioma is induced by COX-2 (cyclooxygenase 2-self-dependent prognostic factor in glioma) expression, through P38 mitogen activation of SP1/SP3 [44]. Some tumor suppressor genes such as phosphatase and tensin homologue (PTEN) can control inflammatory mediator formation [45]. At the early cancer stage, TGF-β (Transforming growth factor) can act as a tumor suppressive factor as they promote apoptosis and an effective anti-proliferative response.

### 3.2. Extrinsic Pathway

Chronic inflammation increases the chances of cancer development and the inflammation in response is mainly triggered by some leukocytes that lead to the formation of inflammatory mediators. They involve exposure to various destructive agents and a broad range of chronic infections, which provoke inflammation and autoimmune conditions [46]. Colorectal cancer is a well-accepted example to explain the connecting link between chronic inflammation and cancer. A patient with Crohn’s disease and inflammatory bowel disease has a higher chance of transforming into colorectal cancer [47]. Approximately 90% of patients consuming tobacco have evidence of lung cancer, while other non-smoking causes of chronic conditions are via air, such as direct contact with airborne atmospheric particulate matters, silicosis, Idiopathic pulmonary fibrosis (IPF), and TB (tuberculosis). All of these are non-smoking causes that are risk factors for lung cancer. The new molecular pathways involve mitochondrial damage and Reactive Oxygen Species (ROS) production, which not only affects DNA damage but also has a role in the activation of oncogenes. The cytidine deaminase family-induced activation act as an RNA and DNA editing enzyme. Heliobacter pylori mediated upregulation of the activation-induced cytidine deaminase (AID) accumulation, causes some major nucleotide alteration inside gastric cells that leads to the development of gastric carcinoma [48]. Abnormal expression of AID in biliary cells generates somatic mutations inside cancer-linked genes including p53 and Emyc. The AID ectopic production might link bile duct inflammation to intensify genetic susceptibility to mutagenesis, which leads to cholangio-carcinogenesis progression [49].

### 3.3. Transcription Factors (NF-kB)

The Nuclear factor kappa B family consists of major five basic subunits in mammals i.e., ReLA (p65), NF-kB1 (p105), NF-kB2 (p100), C-Rel, and RelB each unit requires dimerization in order to bring out transcription activities. The classical (canonical) and alternative (non-canonical) are involved in activating the NF-kB signaling pathway. NF-kB classical pathway activation occurs in response to an inflammatory stimulus such as TNF, InterLeukin-1 beta, and TLR (toll-like receptor). The NF-kB pathway is expressed inside those genes that have a role in cell proliferation, inflammation, survival, and angiogenesis. An alternative NF-kB activation occurs in response to ligand engagement of CD40 (Cluster of differentiation), receptor activator nuclear factor, fibroblast growth factor inducible 14 (Fn14), and lymphotoxin β receptor; all of these are members of the TNF receptor superfamily. The genes that are involved in the regulation of hemostasis of adaptive immunity are regulated by an alternative NF-kB pathway and lymphangiogenesis [50]. This ubiquitous transcription factor is described as a core mediator of immune responses because of its activation by the large diversity of viruses and bacteria. Type 1 macrophages are classical activated and type 2 macrophages are alternative activated [51]. NF-kB has been recognized in gastrointestinal malignancies such as; hepatocellular and colorectal carcinoma. Nuclear factor kappa B consists of 5 ReL proteins family; ReLA, C-ReL, ReLB, P50/105, and P52/100, among all these ReL family proteins only ReLA contains carboxy-terminal transactivation domain necessary for transcriptional activation [52]. The autocrine expression of interleukin 1 alpha and beta in the SUM-149 cell line involve NF-kB activation i.e., necessary for the growth and proliferation [53]. IL-6 and IL-8 inflammatory cytokines are highly secreted during inflammatory breast cancer, and they are among one of the best characterized nuclear factor kappa B target genes [54].

### 3.4. JAK/STAT Pathway

The Janus kinase and STAT signaling pathways are the main mediators for a wide range of physiological responses of cytokine and growth factors at the time of development and hemostasis processes [55]. This signaling pathway is an alternative to the secondary messenger system. JAKs contain tyrosine kinase activities which interact with the cytokine cell surface receptor that further binds to the ligand and receptor that triggers the activation of JAKs, these receptors are activated by receiving signals from interleukin, GF, and interferon. IL-6 and JAK-STAT3 pathways are initiated by genetic alterations in transformed cells. TGF-Beta and NF-kB pathways are regulated by JAK-STAT activation directly and indirectly [56]. The STAT protein determines whether a particular immune response in the tumor microenvironment should be promoted or inhibited. There are seven major STAT family protein members that are encoded by seven different genes: STAT (1, 2, 3, 4, 5A, and 5B) and STAT6. STAT3 has a role in both extrinsic and intrinsic pathways linked to cancer. STAT is activated in malignant cells and has the capability to induce a large number of genes necessary for inflammation. STAT3 and STAT5 are activated proteins, which increase tumor cell proliferation, invasion, and survival. STAT3 plays a dual part in tumor inflammation. It promotes pro-oncogenic inflammation pathways such as NF-kB, and target genes (Interleukin-6) are important STAT3 activators [57]. STAT3 directly interacts with the NF-kB and ReLA in tumor trapping in the nucleus and contributes to the activation of NF-kB in cancer [58]. STAT3 mediates the T-regulating cell accretion/enlargement in tumors. It is essential for the development of TH17 T-cells [59]. In research, it was found that STAT3 have an important role in inflammation-induced adenocarcinoma. It was best observed inside a transgenic mouse model with integrally active glycoprotein (gp130) in epithelial cells [60]. GP130 and STAT3 signaling cause inflammation linked to gastric carcinoma [61].

### 3.5. COX Pathway

Cyclooxygenase-2 (COX) is the key enzyme in eicosanoid biosynthesis, several cancers in humans display upraised levels of prostaglandin (PG) due to the upregulation of COX-2. Approximately 40% of overexpressed COX-2 cases have been found in human breast cancer and pre-invasive ductal carcinoma (in situ lesions) [62]. The transgenic COX-2 overexpression operates the formation of mammary tumors and, in reverse elimination of COX-2, reduces tumor formation in rodent models, especially for breast, skin, and intestinal cancers [63]. The COX enzyme family consists of two members: COX-1 and COX-2 (PG endoperoxide synthase 1 and 2). The upregulation of COX-1 is evoked by various stimuli such as oncogenes, cytokines (IL-1 alpha and beta, interferon-γ, and TNF-alpha), and Growth factors [64]. Both of the COX enzyme family catalyze arachidonic acid conversion to Prostaglandin H2 (PGH2 is successively metabolized to five active prostaglandins; PGI2, PGF2α, PGD2, thromboxane A2(Tx-A2), and PGE2), multiple isomerases which act as a substrate are necessary for the production of eicosanoid products such as Dinoprostone (PGE2), thromboxane-A2 (TXA2), and prostacyclin (PGI2) [65]. COX-2 stimulates angiogenesis, increases the density of microvessels, the expression of pro-angiogenic genes, cell invasion, and suppresses apoptosis. All of these phenomena are regulated by PGE2-activated signaling pathways, which involves MAPAK (Ras Mitogen-Activated Protein kinase), EGFR-Phosphatidylinositol-3 kinase (PI3K), the Mammalian target of rapamycin, Bcl2, Vascular endothelial growth factor (VEGF), Peroxisome proliferator-activated receptor delta (PPAR-δ), and various chemokines and its receptors [66]. The COX-2 expression can be regulated by both levels, transcriptional and post-transcriptional. Transcriptional can be regulated by different transcriptional factors such as NF-kB, Cyclic-AMP response element binding protein, Activator protein 1 (AP1), Peroxisome proliferator-activated receptor—beta and delta (PPAR β and δ) and COX-2, post-transcriptional modulation through Adenine-Uracil rich regions, at 3’ untranslated region binding of the Hu R (RNA binding proteins Hu antigen R) and Tristertraproline at 3’ untranslated region stabilizes and destabilize its mRNA [67]. Many experiments have proven that PGs are directly involved in Inflammatory bowel disease (IBD) and Colorectal carcinoma (CRC); PGE2 controls the pro-inflammatory and tumor-promoting effect of COX-2 in both IBD and CRC [68].

## 4. Cancer-Associated Inflammatory Diseases and Nanotherapy

Inflammatory diseases in which the body’s immune system starts recognizing and damaging its own tissue as an outcome leads to inflammation, when it lasts for a prolonged period of time, it becomes chronic and shuts down the normal process of cancer. Inflammatory diseases such as hepatitis b and c, pancreatitis, and ulcerative colitis (inflammatory bowel disease) are likely linked to the cause of cancer (Table 1). In these diseases, highly reactive molecules are formed by immune cells that lead to DNA damage and may cause cell division. Here, a potent nanotherapeutic approach has been used against inflammation and cancer where nanoparticles were modified to improve their therapeutic ability (Figure 1)and are discussed later in the coming sections (Table 2).

### 4.1. Hepatitis

Long-term infection of hepatitis B and C in the human body can cause Hepatocellular carcinoma (HCC is a chronic viral hepatitis). The hepatitis C and B virus belongs to the Hepadnaviridae family. For all these viruses, human beings are the only natural modes of the host to survive. They are responsible for causing liver cirrhosis, which later transforms into liver cancer. These viruses can lead to DNA damage in the liver cells and develop hepatocellular carcinoma. The hepatitis C virus is the main effective agent of cancer via indirect pathways (chronic inflammation and cell proliferation). In a research report, it was found that the COX protein of the hepatitis C virus has an oncogenic potential when a transgenic mouse model was used for the experiment and found to be effective in inducing hepatocarcinogenesis [69]. Liver transplantation may be successful at an early stage in treating small or slow-growing tumors. Early stages of infection are asymptomatic during the acute phase (6 months). The major cause of hepatitis c infections is mainly by the intravenous use of unauthorized drugs, transplantation of HCV-contaminated cirrhosis, chronic liver disease, hemodialysis, and least frequently intranasal cocaine use [70]. The genetic mutation accumulation in the hepatocytes is necessary for hepatocarcinogenesis [71]. Still, there is no proof that hepatitis is itself oncogenic, HCC may hardly develop in non-cirrhotic hepatitis c infected patients. It is still under controversy whether these viruses may play a direct or indirect role. In a research study, it was found that the presence of the hepatitis B virus gene in chronic HCV-associated liver injuries in a patient was directly involved in the promotion of hepatocarcinogenesis [72]. Interferon therapy could be helpful in promoting survival and in the treatment of HCV-related hepatocarcinoma [73]. Gold NPs are found to be a promising biosensor in detecting hepatitis B virus genes due to its unique property, bio-conjugation. Gold nano rods are considered to be superior and stronger at light scattering and absorption properties in visible and near-infrared regions. Gold NPs have been widely used for the detection of different pathogens, other than the hepatitis virus. The fluorescent-based gold NPs have been applied in plasmodium falciparum heat shock (pfHSP70) protein 70 and a simple immunoassay that was performed for malaria antigen detection, it binds to Au NPs that was CY3B—labeled (Fluorescent quenching of cyanine 3B) recombinant pfHSP70 when released to the solution, it found to show increased fluorescent intensity at a concentration of antigen 8.2 to 23.8 µg mL^−1^ [74]. Immuno-targeted gold nanoshells are used for imaging live HER-2 cells and overexpressed breast cancer cells are labeled with anti-HER-2 nanoshells conjugate [75]. The RNA probe infixed with fluorophores was absorbed on the gold NPs surface in such a way that the probe bound to the targeted RNA and double-stranded RNA complex was released in the solution to reestablish the fluoresce emission [76]. The positive specimen of hepatitis c virus labeled with fluorophores probe hybridized cancer to target hepatitis C virus RNA and the detection of fluoresce was conducted. Gold NPs found promising in detecting hepatitis B surface antigen in an experiment containing monoclonal hepatitis B antibody bound on gold Nano Rod surface to detect hepatitis B surface antigen, after that it was verified by enzyme-linked immunoassay (ELISA) technique [77]. Folic acid targeting moiety with Baicalin loaded selenium NPs (B-SeNPs-FA) of size around 100 nm was found as a succeeding tool to design and synthesized cancer-targeting nanomaterials and used to treat hepatitis B virus-infected patients with live cancer [78]. Nanoparticles can be successfully delivered to the site of hepatocellular carcinoma by passive targeting by the enhanced permeability and retention effect [79]. The synthesized PEGylated PLGA polymer NPs which are co-encapsulated in gemcitabine (GEM) and antisense microRNAs (miRNAs 21), are demonstrated to have increased/fast treatment efficacy in human Hep3B, and HepG2 (Hepatocarcinoma human cancer cells) [80].

**Table 1:** The major inflammatory diseases that are directly associated with cancer and the nanoparticles that play a vital role in these diseases.

Cancer-Linked Inflammatory Disease and Cancer	Nanoparticles	Size	Probe/Target	Action	References
Hepatitis	ZnO NPs	5–50 nm	Zinc NPs binds to viral RNA	Promising in the inhibition of viral replication, when examined on HUH 7 cells against hepatitis C and E viruses	[81]
Hepatitis B	Au NPs	50 nm	Gold NPs-antibody detect hepatitis viral antigen	They target hepatitis B antigen surface to detect the hepatitis B virus present in human serum, via antibody-antigen interaction assays	[82]
Hepatitis C	Amphimedon-Ag NPs	8.22–14.30 nm	NA	They have outstanding anti-HCV, Non-structural protein S drug activity	[83]
Hepatitis B	Ag NPs	10 nm	Silver NPs binds to viral RNA and halts replication	They were found to reduce the formation of extracellular HBV DNA and inhibit RNA and virions when observed on HepAD38 cells	[84]
Hepatic Cancer	Ag NPs	13 ± 1 nm	NA	Inhibition of cytotoxic effects of hepatic cancer at a concentration of 10–200μg/mL on Hep-G2 cells and MCF-7 cells	[85]
Hepatitis	Ag/thiol graphene dots nanocomposite	NA	Riboflavin as a probe	Detection of hepatitis core antigen and use of riboflavin as a redox core probe	[86]
Hepatic cancer	Ag NPs	20–50 nm	NA	They have a potent Cytotoxic effect on human hepatic cancer cells (Huh-7 cells and CHANG) at 0, 5, 20, 40, and 100μg/mL concentration	[87]
IBD	P@QD-MdC NPs	150 nm	Antibody (Anti-MAd CAM-1)	Holds promising outcomes for IBD diagnosis and imagining at an early stage	[88]
IBD	Ginger-derived NPs	~230 nm	Ginger NPs found to have lipids, proteins that binds to cancer cells receptor	They are prominent in the reduction of the effect of acute colitis, repair of intestinal cells, and prevention of chronic colitis-associated cancer	[89]
IBD	Eudragit-Mesoporous Silica nanocomposite	~150 nm	Polymer Eudragit	At an oral dose of 0.2 mg/kg, found to prevent and improve IBD and colitis-associated cancer treatments, reduce the mRNA expression of cytokines (IL-1β, IL-10 and 17), and be effective in the therapy of IBD	[90]
IBD and gastrointestinal disease	Dextran-coated cerium oxide NPs	17.5 ± 0.7 nm and 4.8 ± 1.2 nm (core)	Ceramic oxide encapsulated	For imagining IBD and as a computed tomography agent to give an image of the gastrointestinal tract affected with IBD	[91]
Pancreatic Cancer	Ag NPs	2.6 and 18 nm	NA	Decreased cellular proliferation in PANC-1 cells and higher cytotoxic effect of Ag NPs on human pancreas ductal adenocarcinoma (PANC-1 cells)	[92]
Pancreatic Cancer	Au Nanorods	>6 nm	Polymer (bovine serum albumin) and SiO2 encapsulated	They have applications in bio-imagining and cancer therapy	[93]
Pancreatic Cancer	PEG-ZnO NPs	21.8 ± 0.86 nm	Polyethylene glycol encapsulated	Observe to down-regulate the expression of anti-apoptotic BCL2 and up-regulated pro-apoptotic BAX, and found to have excellent anti-cancer activity on PANC-1 cells	[94]
Pancreatic Cancer	Au NPs	20 nm	Citrate-capped Au NPs	Inhibits proliferation and tumor growth in both pancreatic cancer cells and pancreatic stellate cells	[95]

Abbreviations: IBD-Inflammatory bowel disease, Au NPs (Gold nanoparticles), ZnO NP (Zinc oxide nanoparticles), Ag NP (silver nanoparticles), HUH 7 cells (Human hepatoma-derived), and PLGA-PEG coupled with anti-MAdCAM-1 antibody half-chains and loaded quantum dots (P@QD-MdC NP).

### 4.2. Pancreatic Cancer

The development of malignant cells inside the tissues of the pancreas has the ability to spread to other parts of neighboring tissues inside the pancreas. Inflammation processes have appeared as the main mediators of pancreatic ductal adenocarcinoma (PDAC) development. Chronic pancreatitis has a higher risk of cancer development and progression. PDAC is a highly prevalent neoplastic disease of the pancreas. It is found to be the fourth most frequent cause of death related to cancer worldwide. NF-kB is highly activated in pancreatic cancer. It is known to take part in establishing the communicating link between the tumor and immune cells. NF-kB is capable of regulating inflammatory macrophages directly through the regulation of GDF-15/Mic1 (Growth and differentiation factor 15);, which is highly expressed in the case of pancreatic cancer and serves as an early promoter for the development of cancer. GDF-15 secretion inactivates tumor-infiltrating macrophages (TFM) by negative regulation of TGF-B activated kinase1 (TAK 1) that in response it downregulates NF-kB target genes expression, TNF (Transforming growth factor) and IONOS (Inducible nitric acid synthase) expression, absence causes incapability of macrophages to remove tumor cells [96]. Interleukin-6 promotes the abnormal cell growth on the epithelial tissue surface of the cervix (pancreatic intraepithelial neoplasia (PanIN), TLR-7 and TLR-8 (Toll-like receptor) expression have been shown to increase tumor cell proliferation in human pancreatic cancer and promotes chemoresistance in UICC (Union for International Cancer Control) stage 1st and 4th pancreatic cancer (Panc-1 cancer cell lines). TNF-β was found to play a dual role at an early stage of pancreatic cancer act by promoting apoptosis and inhibiting the progression of the epithelial cell cycle, however, at late stages, it acts as a tumor promoter in tumor invasion, genomic instability, and neo-angiogenesis [97]. TRAIL (Tumor Necrosis Factor related apoptosis-inducing ligand) can induce tumor growth in TRAIL-resistant tumor cells in a syngeneic murine pancreatic cancer model (Murine 6606PDA) [98]. Interleukin-1 β, IL-8, and IL-4 play an important role in acute pancreatitis. IL-1 β induces endoplasmic reticulum stress that releases Ca^2+^ in the cytoplasm and later activates trypsinogen via the impaired removal of damaged organelles (autophagy) during acute pancreatitis (pancreatic acinar cells) [99]. The first research for the early detection of tumor-based Nanoparticles enabled tools (NETs) was first investigated in prostate cancer [100]. The depletion of stroma have been investigated to increase the accumulation of greater molecular weight traces inside the pancreatic tumor through the delivery of PEGylated hyaluronidase (PEGPH20). PEGylated human recombinant PH20 hyaluronidase, induces re-expansion of pancreatic ductal adenocarcinoma blood vessels and increases the delivery of doxorubicin and gemcitabine (chemotherapeutic drug). Gemcitabine, along with PEGPH20, leads to the inhibition of pancreatic ductal adenocarcinoma growth [101]. Theranostic nanoparticles have come out as potent in imaging, therapy, and in early recognition of PDAC. A hyaluronic acid nano-formulation derived Indocyanine green (NanoICG) nanoparticles were employed for intraoperative near-infrared fluorescent detection of pancreatic ductal adenocarcinoma. It was found that due to nanoICG, fluorescent intensity in pancreatic lesions as compared to Indocyanine green NPs alone can do a pathological and hematological analysis of NanoICG proving it to be a noble agent for intraoperative detection of pancreatic tumors when treated on healthy mice demonstrated to have minor toxicity [102]. Hyaluronic acid-mediated iron oxide multifunctional NPs were used for monitoring pancreatic cancer. Hyaluronic acid plays a key part in targeting molecules to identify CD44 surface antibodies. No toxic effects of iron were observed at a concentration up to 100 µg mL^−1^, MiaPaca-2 cells were incubated with HAFe_3_O_4_ NPs at Fe concentration ranging between 5 to 80 µg mL^−1^ at 37 °C temperature and 50% of CO_2_ for about 4 h long. It was observed that cells that were treated with HAFe_3_O_4_ showed an increase in Fe concentration with a sudden decrease in MR signal intensity [103].

**Table 2:** Major nanoparticles that have been widely applied in inflammation and cancer nano-therapies.

Nanoparticle	Size	Role of Action	References
PLGA NPs	350–410 nm	It provides immunotolerance to cancer. It was found to induce anti-tumor therapeutic effects. CD8 T cells secreted interferons at the site of lymph nodes and spleen, and vaccinated mice were treated with PLGA NPs.	[104]
β-Glucan NPs (BG34-*Fe_3_O_4_* conjugated carbon nanotubes)	80–100 nm (Length) 10–20 nm (Diameter)	β-glucan from the cell wall of natural sources such as plants and fungi, have appeared to enhance anti-tumor responses through direct interaction with immune cells such as macrophages and others. It acts as an immune modulator in optimizing tumor microenvironments.	[105]
Anti-PD-L-1 targeted nanoplatform consists of Au-SPIO@PLGA NPs	500 nm	It was found to achieve the promotion of polarization of TAM to M1 (classically activated macrophages) and reverse the cause of immunosuppression by TAM and block the programmed death-ligand 1/Programmed cell death pathway.	[106]
F${\left({ab}^{\prime }\right)}_{2}$ Conjugated–PLGA NPs	500 nm	The efficient and specific T-cell targeting drug delivery binding system in vitro in human cells. In vivo, it allows specific targeted delivery of an inhibitor of TGFβR1 and TLR 7/8 agonist, found to delay the growth of tumors in mice, when delivered via Programmed cell death-1 protein targeting NPs.	[107]
CD44TA-LIP NPs (Liposomes targeting CD44 receptor using Thioaptamers)	204.9	Found to exhibit a host defense mechanism against invading pathogens (TB immunopathogenesis) activate lymphocytes, and provide immunity against tuberculosis in mice.	[108]
Zn-pyrophosphate NPs loaded with photosensitizer pyrolipid (Zn P@Pyro)	NA	It can kill tumor cells, induces apoptosis, and tumor-specific cytotoxic T-cell responses, and disrupt tumor vasculature. It significantly prevents the metastasis of tumors to the lungs of mice.	[109]
PLGA NPs-based vaccine	350–410 nm	It induces specific anti-tumor T-cell responses and activates INF-γ secretion at lymph nodes by activation of CD8+, TRP2 specific T-cells of vaccinated mice bearing melanoma B16 tumors.	[104]
Cytosine-phosphate-guanine coated NPs	NA	It shows rapid accumulation by Antigen-presenting cells and triggers the release of cytokines (IL-10). Induces strong anti-inflammatory responses, enhances TH1/TH2 responses, and eliminates tumor cells.	[110]
Nano-artificial APC iron-dextran coated NPs	50–100 nm	It was found to enhance antigen-specific T-cell proliferation in vitro and inhibition/clearance of tumor growth	[111]

Abbreviations: PLGA-Poly(lactic-co-glycolic acid), TAM (tumor-associated macrophage), CD (cluster of differentiation), Fab (fragment, antigen binding), Au (gold), and SPIO (superparamagnetic iron oxide).

### 4.3. Inflammatory Bowel Disease (IBD)

**Figure 2:**
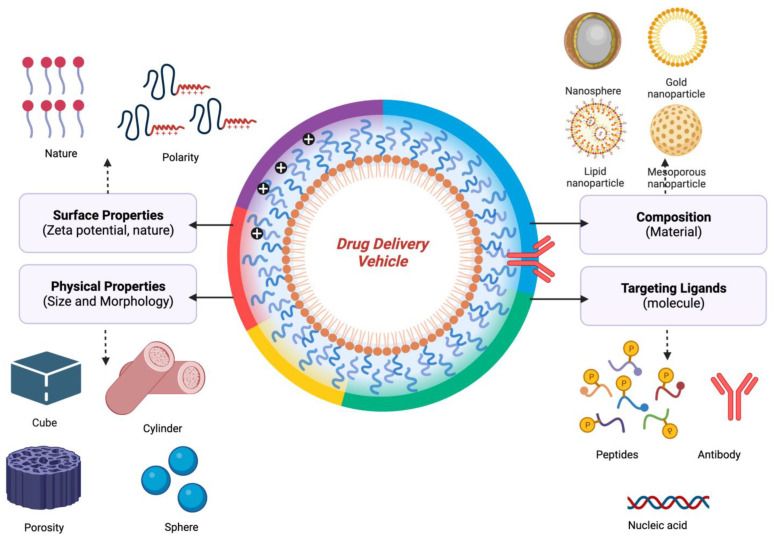
Physiochemical attributes of a drug delivery carrier to consider and improve the therapeutic ability of the drug-loaded drug delivery systems.

It is a type of gastrointestinal disorder caused by long-term chronic inflammation in the digestive tract (intestine) and found to affect all aged groups, most common to those that lie between 15–30 years of age. IBD are of two types: Crohn’s (ileitis) disease and ulcerative colitis, inflammatory infections of Inflammatory Bowel Disease, symptoms of which involve fatigue, abdominal pain, weight loss, and diarrhea. Both Crohn’s and ulcerative colitis are termed idiopathic IBD because of their mechanism of apparent spontaneous origin. Crohn’s disease causes swallowing and irrigation in the digestive tract and entirely affects the thickness of the walls of the bowel. It is also termed ‘Regnal enteritis’, affecting the ileum, the lining of the small and large intestine of the gastrointestinal tract, and is inflamed for a prolonged time. The classification of Crohn’s disease involves Ileocolitis is the most common classification of Crohn’s disease in which inflammation is caused inside the small intestine and some parts of the colon. In ileitis, swelling occurs in the ileum, and there are various other diseases associated with the cause of ileitis (ischemia and neoplasms). Jejunoileitis, the upper half of the jejunum, is marked by the development of patchy areas of inflammation, mainly found in children rather than adults. In gastroduodenal, inflammation occurs at the top part of the small intestine (duodenum) and stomach. Granulomatous colitis affects the colon only. Crohn’s disease is an idiopathic disease, yet there are certain factors that may increase its risk of development, for example, autoimmune disease, genes, smoking, and embracement of guts bacteria. Ulcerative colitis causes inflammation and sores inside the innermost large intestinal lining (colon) and rectum of the digestive tract. It is classified into different types according to the site of infection involves ulcerative proctitis, a form of ulcerative colitis that contains fine ulceration in the inner mucosal lining of the large intestine (area closed to rectum) it only affects the lowest part of the colon. Rectal bleeding is the only sign of this disease occurrence. Proctosigmoiditis affects the sigmoid colon and rectum, it is a mild type of ulcerative colitis, and infection starts from the rectum and goes into the lower end of the colon near the sigmoid colon. The sign of this disease occurrence can be mostly felt on the left side of the abdomen. Pancolitis causes inflammation of the whole part of the colon, which is why it is also called universal colitis/total colitis. Left-side colitis/distal ulcerative colitis causes inflammation only on the left part of the colon, inflammation extends from the rectum to the splenic flexure (the site where the colon bends near the spleen). Ulcerative colitis can sometimes cause life-threatening medical complications. It is an incurable disease, and therapies and treatment can only reduce its signs and symptoms. They can only suppress the effect of cancer for a long time. Nanomedicines are widely applied for the treatment, imagining, and diagnosis of IBD and colitis-associated cancer. Edible ginger-derived NPs (GDNPs 2), at a size of 230 nm, were found to be effective in colon targeting. These NPs consist of high lipids levels, few protease, microRNA, and bioactive constituents of ginger (6-gingerol and 6-shogal) found to be effective in acute colitis, prevent colitis-linked cancer, and chronic colitis. GDNPs 2, when orally administered, increases the proliferation of intestinal epithelial cells, and reduces proinflammatory cytokines and anti-inflammatory cytokines when used in a colitis mouse model [89]. The Up-conversion nanoparticles are newly developed lanthanide (Ln) doped nano-crystals that uses light-triggered luminescent probes in drug delivery. It converts long near-infrared wavelength emission, preferably used for the treatment of colorectal cancer [112]. Ln 3+ ions doped inorganic crystals used as sensitizers and activators. Its main advantage is that it does not suffer photo bleaching and gives high photochemical stability and gives early identification of patients inclined to develop inflammatory bowel disease (e.g., colorectal cancer) [113]. The titanium dioxide NPs of size range between 35.76–78.17 nm, were synthesized by *Bacillus tequilensis* on expression of clbB and clb N genes of Escherichia coli bacterium that is responsible for colorectal cancer. Titanium dioxide NPs were inspected by X-ray diffraction, SEM, infrared spectroscopy, and RT-PCR. Real time-PCR exhibited 20 folds depletion in the expression of clb B and clb N genes and showed lower toxicity [114]. The green synthesized Ag NPs using the *Artemisia tournefortiana* extract (plant extract) on HT29 (human colon cancer) and HEK 293 (Normal cell) were treated with different concentrations of synthesized Ag NPs for 24 h. Silver NPs were found to extensively decrease the viability of the cells 8n dose and time-dependent manner. Half maximal inhibitory concentration value of Ag NPs was found as 40.71 and 61.38 mg/mL exhibited a more cytotoxic effect on HT29 colon cancer cells than HEK 293 normal cells [115]. The capped Au NPs with polyethylene glycol 9000 were found to be superior for anti-cancer activity at a half maximal inhibitory concentration (687.44 µg/mL) against MCF-7 cells (Breast cancer cell lines) and anti-inflammatory activity at Half maximal inhibitory concentration 287.177 mg/L [116]. Heat shock protein (HSP 90) targeted therapy has come out as an auspicious strategy for treating colitis-associated cancer and ulcerative colitis (UC) in murine mouse models (mouse colon 26 cells) when administered orally [117]. NPs are considered a wonderful vehicle in drug delivery because of their properties such as type (organic and inorganic NPs), size, surface charge, nature, morphology, composition, and the targeting ligands (Figure 2). They help in the delivery of drugs at destined sites, boost immune responses, and efficacy of the treatment. Rapidly penetrate deep down the tumor cells and help in the clearance of tumors by site-specific targeting.

## 5. Conclusions

Inflammation is the body’s normal response to an infection but over time it may give rise to fatal consequences when it lasts more than usual/normal (chronic) inside the body, transforming into cancer that can be life-threatening. Many factors and signaling pathways are involved in this process. Chronic inflammation is linked to cancer causes; cell proliferation, cell mutation, and lead to DNA damage. Nanoparticles are promising not only for the treatment, but also play a vital part in the early imagining, detection, treatment, and diagnosis of cancer and inflammatory diseases. Diseases such as inflammatory bowel disease, hepatitis, and pancreatic cancer, all these diseases cannot be cured easily without any surgery but by the rapid action of NPs, they can be diagnosed at early stages. Nanoparticle treatment helps in suppressing its fatal consequences for a certain period of time. Nanotechnology can be friendly to those people who cannot afford such high-cost treatments and therapies.

## Data Availability

Not applicable.
